# Shedding Rates and SeroPrevalence of *Brucella melitensis* in Lactating Goats of Shahrekord, Iran

**DOI:** 10.5812/jjm.9394

**Published:** 2014-03-01

**Authors:** Azizollah Ebrahimi, Jalal Sheykh kanluye Milan, Mohamad Reza Mahzoonieh, Khadijeh Khaksar

**Affiliations:** 1 Institute of Zoonotic Diseases, Shahrekord University, Shahrekord, IR Iran; 2 School of Veterinary Sciences, Shahrekord University, Shahrekord, IR Iran; 3 Office of Veterinary Organization, Shahrekord, IR Iran

**Keywords:** Goats, Brucellosis, *Brucella melitensis*, Milk, Vagina, Serology

## Abstract

**Background:**

Brucellosis remains a major worldwide zoonosis. Caprine brucellosis is a significant problem for both public health and animal production. *Brucella melitensis* causes disease in goats, sheep, humans, and occasionally cattle. Transmission is by ingestion or contact with infected materials, vaginal discharge, or milk.

**Objectives:**

The current study aimed to determine the rate of *B. melitensis *seropositives and its probable shedding in lactating goats from flocks in Shahrekord district, Iran.

**Materials and Methods:**

In the current study, 1080 samples of milk, blood and vaginal swabs of 360 lactating goats (three samples from each animal) were randomly collected from 12 flocks in Shahrekord district. Serums from blood samples were examined by Rose Bengal plate (RBT) test and the titre of positives determined by tube agglutination test (TAT). Vaginal swab and milk (cream and sediment) samples were cultured on Brucella agar. *Brucella *spp. suspected pure cultures were incubated in the same conditions and then examined by Modified Zeil-Nelson (MZN) staining, oxidase and catalase tests. Positive isolates were examined by PCR.

**Results:**

Out of 360 serum samples, 50 (13.9%) were positive by RBT, and six (1/66%) were positive by TAT. Culturing of milk and vaginal samples lead to isolation of 12 (3.33%) and 10 (2.77%) *Brucella* spp. suspected colonies, respectively. The PCR examinations of these isolates showed that ten (2.77%) milk and 6 vaginal swab samples (1.66%) belonged to *B. melitensis* species. Eight goats (2.22%) had positive results in RBT, culture and PCR examinations, simultaneously.

**Conclusions:**

The regional distribution of caprine brucellosis and shedding of *B. melitensis* through vaginal secretions and milk secretions of lactating goats indicated that 50% and 83.33% of the goat flocks contained vaginal and milk shedders, respectively.

## 1. Background

Brucellosis is an important zoonosis causing debilitating disease in humans. Brucella species and their principal farm animal hosts are *Brucella abortus* (cattle), *B. melitensis* (goats), *B. suis* (pigs), and *B. ovis* (sheep) (1). In general, the principal manifestations of brucellosis are reproductive failures such as abortion or birth of unthrifty newborn in the female, and orchitis and epididymitis with frequent sterility in the male. Caprine brucellosis is a significant problem for both public health and animal production. *B. melitensis* cause disease in goats, sheep, humans, and occasionally cattle (1).

Transmission is congenital or by ingestion or contact with infected placenta, vaginal discharge, or milk. Infected goats, whether aborted or given birth normally, discharged a large number of* Brucella* spp. in their uterine exudates and placenta (2). The majority of goats that are infected during pregnancy will excrete the organism in their milk in the subsequent lactation, and many will excrete it in all future lactations (2). Isolation of the organism from the aborted fetus, vaginal mucus, or milk is the common laboratory procedure applied in diagnosis. The conventional serological tests for the diagnosis of *B. melitensis *are agglutination, CFT, and the Rose Bengal or card tests. Conventional serological tests will not differentiate infection with different species of *Brucella*. The Rose Bengal test has excellent specificity and high sensitivity (3).

PCR assay is a rapid and sensitive technique for diagnosis of brucellosis compared to serum agglutination test (SAT) method. However it is more valuable when coupled with conventional methods (4). The prevalence of brucellosis is increasing in many developing countries (5). Several authors have reported the prevalence of brucellosis in both animals and humans in various parts of Iran (6-9). However, information is scarce on brucellosis in goat flocks in Iran. The current study was conducted to evaluate status of caprine brucellosis by serological tests and also isolation and identification of *B. melitensis* from milk samples and vaginal swabs of lactating goats in Shahrekord district, west of Iran.

## 2. Objectives

The current study aimed to determine the rate of *B. melitensis *seropositives and its probable shedding in lactating goat flocks in Shahrekord district, Iran.

## 3. Materials and Methods

### 3.1. Sample Collection and Serological Tests

From March to June 2012, 1080 samples of milk, blood and vaginal swab of 360 lactating goats (three samples from each animal) were randomly collected from 12 flocks rearing in different regions of Shahrekord. Based on owners statements all flocks had been vaccinated against brucellosis, however, some owners feared to say that their flocks were unvaccinated in case of ignored vaccination. Ten flocks composed of both sheep and goat populations. The samples transferred to the microbiology laboratory of institute of zoonotic diseases, Shahrekord University in sterile tubes that in the case of vaginal swabs contained 2 mL of Tryptic Soy Broth (TSB) (Merck, Germany). Serums from blood samples were examined by Rose Bengal Plate test (RBT) and the titers of positive samples were determined by tube agglutination test (TAT). RBT and TAT antigens were purchased from Razi Serum and Vaccine Research Institute and Behine Parvar Parsian Institute, Tehran, respectively.

### 3.2. Milk and Vaginal Swab Cultures

Samples of vaginal swab and milk (cream and sediment) were cultured on Brucella agar plates that contained 5% bovine blood, bacitracin (25 IU/mL), Polymyxin B (6 IU/mL) and incubated for 72 hours at 37˚C (10). *Brucella* suspected colonies were pure cultured and after incubation in the same conditions and then examined by modified Zeil-Nelson (MZN) staining and oxidase and catalase tests (10). Modified Zeil-Nelson, oxidase and catalase positive isolates were cultured in TSB medium and stored for further examinations.

### 3.3. PCR Examination

The crude DNA was extracted by Quigley and Holmes method (11) from suspected *Brucella* spp. In brief, a few colonies of bacteria were suspended in 1 mL of sterile water and boiled for 15-40 seconds and after a low-speed centrifugation the supernatants were used as a DNA source. The applied *B. melitensis *specific primers have been previously described by Bricker and Halling (12). 

The sequences of the forward primer was 5ʹ-AAATCGCGTCCTTGCTGGTCTGA-3ʹ (*B. melitensis *specific primer) and the reverse was 5ʹ-TGCCGATCACTTAAGGGCCTTCAT-3ʹ (IS711-specific primer). PCR assay was performed in a final volume of 25 μL mixture containing: 10 times PCR buffer (50 mM KCl, 10 mM Tris-HCl pH = 8.3), 1.50 μL MgCl_2_ (50mM), 0.25 of each deoxynucleotide triphosphate (10 mM), 1 μL of each primer, Taq DNA polymerase (5 U/μL, Cinnagen, Iran) and 2 μL of bacterial DNA as the template. The amplifications were carried out in a Corbett thermo cycler, as follows: initial denaturation at 94˚C for 1 minute followed by 35 cycles of 95˚C for 1.15 minutes, 55˚C for 2 minutes and 72˚C for 2 minutes with a final extension at 72˚C for 5 minutes.

The products were analyzed by electrophoresis through a 1.5% (w/vol) agarose gel, after which the gel was stained with 0.5 μg/mL Ethidium bromide, and DNA fragments were visualized by a UV Transilluminator. Positive and negative controls of PCR were included in all experiments. *B. melitensis* Rev1 strain (Razi Institute, Iran) was used as the positive and deionized double distilled water as the negative control. Negative control, containing all the reagents but lacking template DNA was processed exactly as previously described to monitor for contamination with Brucella DNA, which was negative in all experiments.

## 4. Results

**Figure 1.**
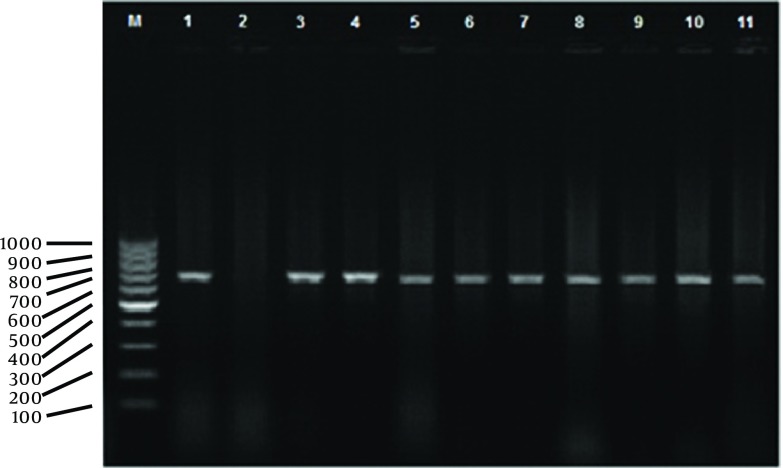
PCR Products Amplified From *Brucella* spp Suspected Colonies. Genomic DNAs From Bacterial Colonies Were Tested in the PCR Assay as Described in the Text Lane M, Marker 100 bp (base pair) DNA Ladder (Fermentas, Slovenia), lane 1, Positive Control (*B. melitensis*, Rev 1 strain, Razi, Iran), lane 2, Negative Control (Water), lanes 3-11 Samples

Overall, out of 360 serum samples, 50 (13.9%) were positive by RBT out of which six (1.66%) and 19 (5.27%) were positive (titers of 1.40 or higher) and suspicious by TAT, respectively. Culturing 360 milk and 360 vaginal samples led to isolation of 12 (3.33%) and 10 (2.77%) suspected *Brucella* spp. colonies, respectively (22 isolates, in total). Details are summarized in Table 1. Eight goats (2.22%) were positive in RBT, culture and PCR examinations, simultaneously. Test results of these isolates by PCR method showed that 10 (2.77%) milk and six vaginal swab samples (1.66%) belonged to *B. melitensis*, Figure 1. In six out of 12 studied flocks (50%) there were at least one vaginal excretors while for milk this number was 10 (83.33%), details are summarized in Table 2. 

**Table 1.** Results of Rose Bengal Plate Test, Milk Culture and PCR of Suspected Isolated *Brucella* spp. Colonies From Milk and Vaginal Swab Cultures of 360 Lactating Goats in Shahrekord District ^a^, ^b^

Methods	Vaginal Swab		Milk		Serum	
	Positive, No. (%)	Negative, No. (%)	Positive, No. (%)	Negative, No. (%)	Positive, No. (%)	Negative, No. (%)
**Culture**	10 (2.77)	350 (97.23)	12 (3.33)	348 (96.67)		
**RBT**					50 (13.9)	310 (86.1)
**TAT**					6 (1.66)	44 (12.22)
**PCR**	6 (1.66)	4 (1.11)	10 (2.77)	2 (0.55)		

^a^ Abbreviations: PCR, polymerase chain reaction; RBT, Rose Bengal test; TAT, tube agglutination test.

^b^ TAT of 50 RBT positive samples; PCR of positive cultures

**Table 2.** Number of Goats Excreting *Brucella melitensis* in Flocks of Shahrekord District ^a^

Flock Numbers	PVS	PM
**1**	1	1
**2**	1	1
**3**	0	1
**4**	0	1
**5**	1	0
**6**	0	0
**7**	0	2
**8**	0	1
**9**	0	0
**10**	1	1
**11**	2	1
**12**	0	1

^a^ Abbreviations: PM, positive milk samples; PVS, positive vaginal swabs.

## 5. Discussion

Brucellosis remains a major worldwide zoonosis (13). The study results indicated that the shedding rates of *B. melitensis* by lactating goats in Shahrekord, west of Iran, was 2.77% in milk samples and 1.11% by vaginal excretions. This rate of shedding of *B. melitensis* by goat populations in the district could potentially lead to public health and veterinary problems. The study results also showed that 50% and 83.33% of the flocks contained vaginal and milk shedder goats, respectively. Also the possibility of sheep shedders in these flocks should not be ignored. Pasteurization and boiling of milk and milk products may limit transmission of *B. melitensis* to human and healthy animals, but the story for vaginal excretors is different.

The selected animals were not just after lambing, so in this case disinfection of lambing sites does not seem to have a considerable effect on limiting the distribution of the bacteria to different environments. Although this method is certainly a major program to control animal brucellosis. Moreover, most of the flocks in this district have a seasonal migration pattern to neighboring provinces such as Khuzestan and Fars, which can contaminate other parts of the country. It seems that to control these shedders, development of detection and slaughter policies are the best choice but in the case of small ruminants, in Iran such policies have not been followed due to some technical and economic problems.

Moshkelani et al. (6) reported high isolation rates (16.4%) of *B. melitensis* from aborted goat fetuses in Chaharmahal-va-Bakhtyari province, indicating the significant role of *B. melitensis* in goat abortions in the district. From east of Iran (Birjand city) Bokaie et al. reported a 3.4% seroprevalence for sheep and goat brucellosis (14). Zowghi and Ebadi (15) isolated *B. melitensis *from 0.24 and 3.4% of bovine tested milk and aborted fetus samples, respectively; the report emphasized on the role of *B. melitensis* in bovine infection, excretion in bovine milk and bovine abortions. The current study results are in line with those of Esmaieli et al., (16) indicated that 2.1% of sheep and goats in Iran are suffering from brucellosis. The current study’s serological tests, RBT and TAT, showed 13.9% and 1.66% seroprevalence for caprine brucellosis, respectively. The difference between the two tests may be due to inactive brucellosis or vaccinal antibodies detected by RBT but ignored by TAT.

There is one report from Sarab city (17), north west of Iran, that showed a 5% seroprevalence for goats in this region. The difference in these reports might be from differences in the study design, duration, variation among infected flocks, stage of infection, and variation in the sensitivity of the tests used. *Brucella* spp. isolation is influenced by factors such as highly fastidious growth requirement, a fewer number of viable organisms in the specimen, delay in transportation and earlier treatment with chemotherapeutics (18). The current study found that many goat farmers seemed to be unaware of the importance of hygiene and other managemental factors to prevent *Brucella *spp. transmission.

In conclusion the current study indicated regional distribution of caprine brucellosis and shedding rates of 1.1% through vaginal and 2.77% through milk secretions of lactating goats, and that 50% and 83.33% of the flocks contained at least one vaginal or milk shedders, respectively. Moving these flocks should be controlled by appropriate regulations. Infected animals must be detected and slaughtered and all goats should be vaccinated against brucellosis.
